# Plastic microfibre ingestion by deep-sea organisms

**DOI:** 10.1038/srep33997

**Published:** 2016-09-30

**Authors:** M. L. Taylor, C. Gwinnett, L. F. Robinson, L. C. Woodall

**Affiliations:** 1 Department of Zoology, University of Oxford, Tinbergen Building, South Parks Road, Oxford OX1 3PS, UK; 2 Department of Forensic and Crime Science, Staffordshire University, Stoke-On-Trent ST4 2DF, UK; 3 School of Earth Sciences, University of Bristol, Bristol, BS8 1RJ, UK; 4 Department of Life Sciences, Natural History Museum, London SW7 5BD, UK

## Abstract

Plastic waste is a distinctive indicator of the world-wide impact of anthropogenic activities. Both macro- and micro-plastics are found in the ocean, but as yet little is known about their ultimate fate and their impact on marine ecosystems. In this study we present the first evidence that microplastics are already becoming integrated into deep-water organisms. By examining organisms that live on the deep-sea floor we show that plastic microfibres are ingested and internalised by members of at least three major phyla with different feeding mechanisms. These results demonstrate that, despite its remote location, the deep sea and its fragile habitats are already being exposed to human waste to the extent that diverse organisms are ingesting microplastics.

There appears to be no environment on Earth that has escaped plastic pollution. Indeed, despite the distance from land, plastics are ubiquitous in remote marine environments, including polar regions^1^,^2^. These plastics are known to cause impacts to terrestrial and marine ecosystems both at the macro- and micro-scale. For example, ingestion of plastic debris or entanglement has been recorded in 44–50% of all seabirds^3^, sea snakes, sea turtles (all species), penguins, seals, sea lions, manatees, sea otters, fish, crustaceans and half of all marine mammals^3^,^4^,^5^. Ingestion can block the digestive tract, damage stomach lining and lessen feeding, all leading to starvation (reviewed in ref. 4). Of growing concern are microplastics (typically defined as <5 mm^6^). The large surface area-to-volume ratio of microplastics, compared to macroplastics, means they concentrate persistent organic pollutants which can be up to six orders of magnitude more contaminated than ambient seawater^7^ and absorb metals^8^,^9^. The subsequent transfer of such pollutants and additives from microplastics to marine organisms has been confirmed under experimental conditions^6^,^10^,^11^. However, the ecological effects on marine organisms in the wild is understudied and not yet conclusive^12^.

It has already been shown that microplastics are ingested by large pelagic marine organisms such as filter-feeding salps^13^, tuna^14^, and whales^15^,^16^. However, only a few observations have demonstrated that microplastics are being ingested in natural settings by benthic organisms, mostly in shallow coastal waters. These observations include organisms with different feeding strategies. Organisms such as the detritivorous and predatory lobster^17^,^18^ and shrimp^19^ presumably consume microplastic passively with prey (or in the prey itself) and/or sediment. Deposit-feeding lugworms^20^ likely consume microplastic that are within sediment, and suspension/ filter-feeding mussels likely take in plastics that are suspended in sea water^20^. In a natural setting microplastics have been found in the stomach^21^,^22^, oral^23^ and ventilation areas^22^,^24^ of shallow-water organisms; and on the outer surface of deep water octocoral^25^. Laboratory studies suggest that benthic and invertebrate taxa, including corals^23^, copepods^21^, zooplankton^26^, crabs^24^,^27^, molluscs^6^, sea cucumbers^28^, scallops^29^, barnacles^30^, oyster^31^, lugworms and polychaetes^10^,^32^ will ingest microplastics if they are introduced under experimental conditions. The effects across this range of organisms included reductions in fecundity^21^, lower feeding rates^26^, enhanced susceptibility to oxidative stress, reduced ability to remove pathogenic bacteria^10^, reduced feeding activities^27^,^33^, reduced energy reserves and balance^27^,^32^, and decreased lysome stability^22^. The results of these studies are not yet conclusive, but the sum of existing laboratory experiments, most of which use microbeads and not microfibres, highlight the detrimental effects of microplastics in a broad range of benthic taxa and the importance of considering organism biology e.g. low metabolism^27^, feeding method^33^ and behaviour.

The ultimate fate of microplastics that reach the deep-sea realm is also not as well studied as in shallow waters. Recently, microplastics have been identified in the deep and abyssal oceans^25^,^34^,^35^, the largest marine habitat on the planet. These studies suggested that deep-sea microplastics are already being found in similar concentrations as intertidal and shallow sub-tidal sediments^25^. The rate of accumulation of microplastics in the deep sea has not been researched, neither has impacts on deep-sea organisms. However, given the ever-increasing plastic load reaching our oceans^36^, and that a large portion of plastics will likely eventually end up on or buried in the seafloor, the potential is there for an unseen pervasive impact on deep-marine ecosystems.

As yet, there have been no studies to establish whether organisms of the deep sea will ingest microplastics or what the impacts may be. Indeed, impact studies will be even more challenging in the deep sea than for shallow marine organisms given the logistical constraints of studying life hundreds to thousands of metres beneath the waves. Here we use specimens collected from two deep submergence research cruises to two different ocean basins to show for the first time that deep-sea organisms from at least three different phyla are ingesting and /or internalising plastic microfibres.

## Results

**Figure 1:**
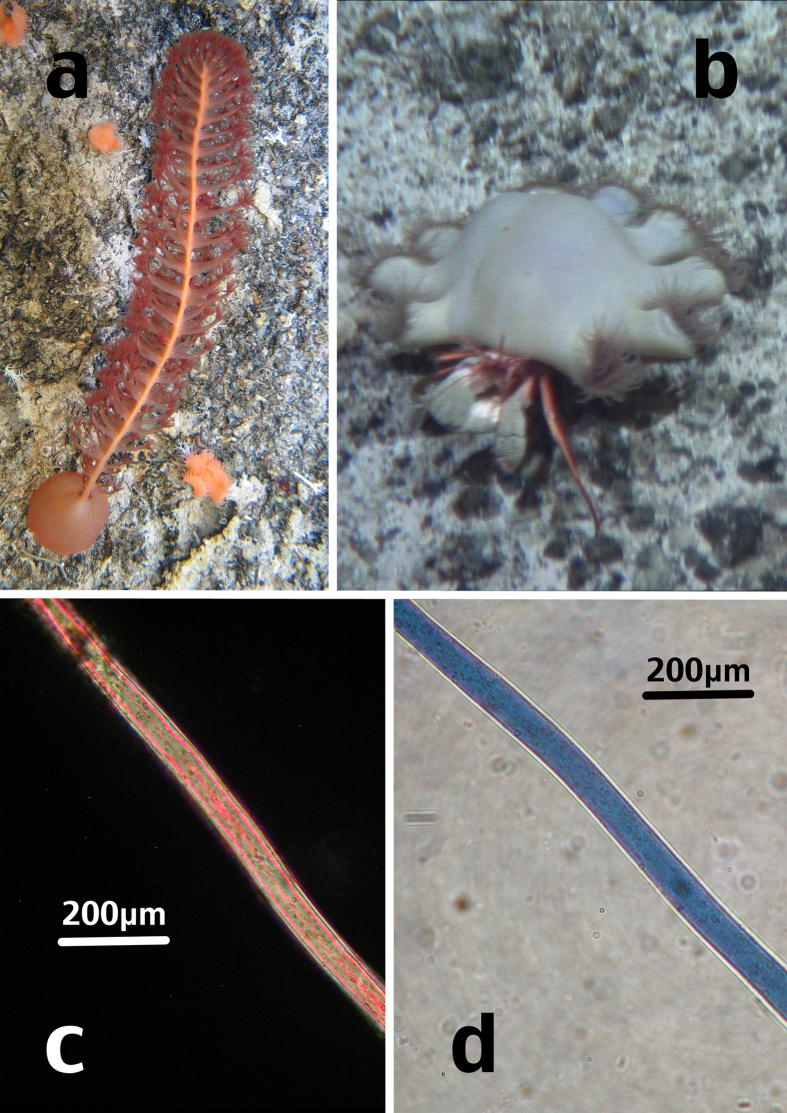
Images of specimens *in situ* (**a**,**b**) and close-up images of microplastic fibres exhibiting their interference colours (used to aide classification) under cross-polarised illumination (**c**) and under plain polarised light (**d**); (**a**) sea pen, JC066-3717; (**b**) hermit crab with zoanthid symbionts, JC066-702; (**c**) polyester microfibre, JC066-702-09; (**d**) acrylic microfibre, JC066-702-10. Images (**a**,**b**) taken by MLT. Images (**c**,**d**) taken by CG.

All microplastics found in this study were microfibres (e.g. Fig. 1c,d), not microbeads. All fibres (15) were of different classes and were constructed from 1 of 5 different materials (modified acrylic, polypropylene, viscose, polyester, and acrylic). This variability between samples provides evidence that there was limited or no contamination as there was no consistency in microfibres across expedition or organism. In a wider study of microfibres from the deep-sea^37^ just 2 of the 52 classes of plastics found in samples were found in contamination monitoring efforts; this minimal overlap makes it unlikely, given the same protocols were followed, that the microfibres from within organisms presented here are the results of contamination. In addition, following Woodall *et al*.^37^ clean room protocols, monitoring of potential laboratory contamination using dampened filter papers indicated that there were no synthetic fibres contaminating the laboratory used for dissection.

**Figure 2:**
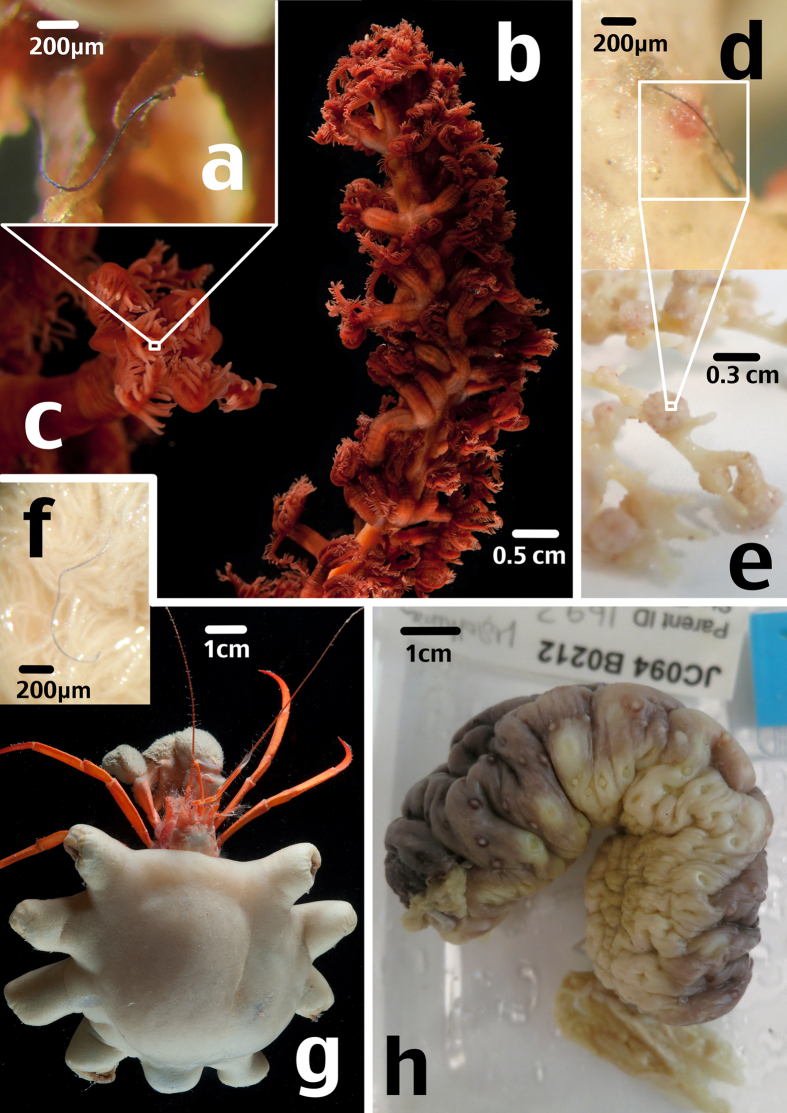
Organisms found to have ingested microfibres and microfibres *in situ*; (**a**) blue microfibre from mouth area of sea pen polyp (**b**) sea pen, JC066-3717; (**c**) example sea pen polyp; (**d**) black mirofibre embedded in surface of zoanthid; (**e**) zoanthids on bamboo coral skeleton, JC094-767; (**f**) blue microfibre on feeding maxilliped of hermit crab; (**g**) hermit crab, JC066-702, with symbiotic zoanthid; (**h**) sea cucumber, JC094-212. Images taken by MLT.

Plastic microfibres were found on and inside six of the nine organisms examined (Table 1), including examples of taxa from the phyla Cnidaria, Echinodermata and Arthropoda. Specifically microfibres were found inside either oral areas (seapen tentacles and upper mesentry, JC094-3717, Figs 1a and 2a–c), feeding apparatus (hermit crab maxilliped, see Fig. 2f,g; JC066-702), symbiotic zoanthid tentacles (zoanthid on hermit crab, JC066-702, Figs 1b and 2g), gill (squat lobster, JC094-771) or stomach areas (sea cucumber, JC094-212, Fig. 2h). Similar to^24^, one of the Crustacea studied (squat lobster, JC094-771) had microfibres in the gut and in the ventilation/gill areas. Microfibres were not found inside zoanthids that were covering a bamboo coral skeleton (Fig. 2d,e; JC094-767) but were found externally. No microfibres were found in or associated with the anemone, armoured sea cucumber or other octocoral investigated.

## Discussion

**Figure 3:**
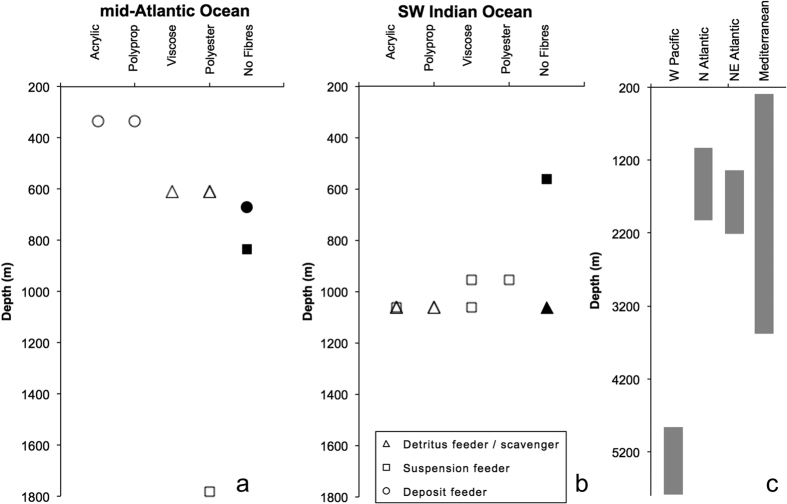
Microplastic presence by material and depth, (**a**) mid-Atlantic data from JC094; (**b**) SW Indian Ocean data from JC066; (**c**) depth of all other known deep-sea microfibres found in sediments represented by grey bars.

Microfibres inside deep-sea organisms were found from 334–1783 m depth in the equitorial mid-Atlantic and 954–1062 m in the SW Indian Ocean (Fig. 3). Previous studies have found microfibres in sediments down to 2000 m in the subpolar North Atlantic, 2200 m in the NE Atlantic, 3500 m in the Mediterranean and 5768 m in the West Pacific^35^. Most deep-sea organisms rely either directly or indirectly on the supply of organic detritus from the euphotic zone, often called ‘marine snow’. Our confirmation of biological integration of microplastics makes recent evidence of a shift towards smaller plastic size categories, equivalent to the ‘marine snow’ size^38^, something now particularly relevant for deep-sea organisms.

In the few instances where they have been studied in deep sea sediments, microplastics occur in similar concentrations as in inter-tidal and shallow sub-tidal sediments^25^. Enders, *et al*.^39^ recently modelled microplastic distribution to 250 m depth but there is no raw data from deep-sea water columns on the High Seas. We assume that microplastics in sediment represent a vertical accumulation from falling ‘marine snow’^25^. We observed that the suspension-feeding anemone, armoured sea cucumber and octocoral had no microfibre load, although fibres were found inside the suspension-feeding sea pen and zoanthid from the SW Indian Ocean (Table 1, Fig. 3). By contrast fibres were found in all predatory, deposit and detritivore feeders examined. If this general observation (albeit based on very few samples), of filter-feeders having lower microplastic loads, holds true more widely, the implication is that deposit-feeding organisms may be more vulnerable to microplastic ingestion than suspension feeders. Of course, load depends on a wide range of factors, such as an animal’s ability to avoid microplastic ingestion, any size or shape-selection of food particles etc. and the abundance and density of microplastics found in an organism’s environment. Knowledge of background microplastic load, systematic surveys with multiple replicates of sediment, seawater collections and sampling of deep-sea organisms across a range of feeding strategies would be required to test if feeding strategy alone impacts organism vulnerability to microplastic ingestion.

Despite microfibres being the majority of microplastic pollution^40^,^41^, including in the deep-sea^25^,^35^, most feeding experiments that have been undertaken thus far use microbeads and plastic shavings, with a few exceptions, Hämer, *et al*.^42^, Watts, *et al*.^27^, Au, *et al*.^43^. Our study shows for the first time that deep-sea organisms are ingesting microfibres in a natural setting, thus we suggest that experimental designs using fibres are needed to determine the potential long-term impact of microplastics for both shallow and deep marine organisms.

The range of plastic microfibres found ingested/internalised by organisms studied here included modified acrylic, polypropylene, viscose, polyester, and acrylic. Polypropylene has been found to adsorb PCBs (polychlorinated biphenyls), nonylphenol and DDE, an organochlorine pesticide^7^. Polyethylene, a type of polyolefin fibre whose chemical composition in part is the basis of some polyester fibres (e.g. polyethylene terephthalate), has been found to adsorb four times more PCBs than polypropylene^44^. Polypropylene has also been found to adsorb a range of metals in a marine environment; the concentrations of most of these metals did not saturate over a year period suggesting plastics in the oceans for long time periods accumulate greater concentrations of metals^9^.

Chemical contamination experiments are rare in the marine environment, and often present unrealistic experimental scenarios^45^. Yet with the chemical ingredients in 50% of plastics listed as hazardous (United Nations’ Globally Harmonized System of Classification and Labelling of Chemicals) such issues maybe just the start of long-term ecological and health problems associated with waste plastics in the environment^46^; impacts that have not been looked at in many marine animals^6^,^10^,^11^ and no deep-sea animals as yet.

Of course, ingestion, and any subsequent biological impacts, depend on many factors^32^ including characteristics of the microplastics themselves, such as size, shape, density, abundance (as seen in shallow water sea cucumbers^28^), colour, and importantly, differential adsorption of harmful substances^7^, as well as organism physiology, ecology and behaviour; this also includes whether microplastics accumulate in the organism, feeding method and/or prey of organisms, and where microplastics accumulate, or are egested and/or translocate within the organism. A final factor is whether transfer of the microplastic up the food chain is a possibility. All of these facets of the microplastic biological impact problem are relevant to deep-sea organisms however as science knows less about deep-sea biology and ecology (as there are fewer experimental opportunities in this challenging environment) these aspects of marine pollution will be relatively difficult to pursue.

Shallow-water experiments have found microplastic bioaccumulation e.g. lobster^17^, mussel and oyster^47^. Given that our data are a snapshot of the fibres within six organisms we cannot determine whether microfibres are bioaccumulating. Five microfibres was the most found within one organism (the hermit crab, JC066-702) and not in a ball as was seen in the lobster *Nephrops*^17^ and crab, *Carcinus maenas*^27^. This could suggest that microplastics are transient within the organisms studied. Or, this could be indicative of low densities of microplastics in the deep-sea feeding areas of organisms studied here, or that microfibres have different residency times to other more intensively studied microplastics (e.g. microbeads), or that the organisms here have different gut residency times to other organisms studied. It may also be that certain feeding strategies convey less suspectibility to microplastic bioaccummulation. Given the low number of organisms it was possible to sample here, and without concurrent environmental sampling, the link between background microplastic densities and microplastic abundance within organisms is not possible to establish.

## Conclusions

Studied organisms have a range of feeding mechanisms, from suspension feeding (sea pens, zoanthids, anemones, barnacles, armoured sea cucumbers) to deposit feeders (sea cucumbers), detritivores and predators (hermit crabs, squat lobsters). Given the breadth of feeding strategies found in deep-sea organisms and their reliance on ‘marine snow’ (which is the same size fraction as microplastics), and evidence of ingestion in shallow-water counterparts, there is a high likelihood of microplastic ingestion across a wider range of taxa than presented here. However, without the context of environmental sampling of microplastics (water and sediment) or investigations into the impacts of the chemicals ingested, it is not easy to understand the impact microplastic presence will have on biology, and subsequently ecology, of deep-sea organisms. Broadly, the important individual organism effects of microplastic ingestion are being investigated (albeit mostly with microbeads rather than the more commonly found microfibres) but, given the ubiquity of microplastics in our marine environments, research should start considering population and ecosystem level effects^48^ such as differential age/cohort survival causing demographic shifts, food/prey shifts, hazard to human foods, taxa specific vulnerability etc; this is a difficult task in any marine environment, most especially the deep-sea, regardless it is still an important challenge to undertake.

## Materials and Methods

**Figure 4:**
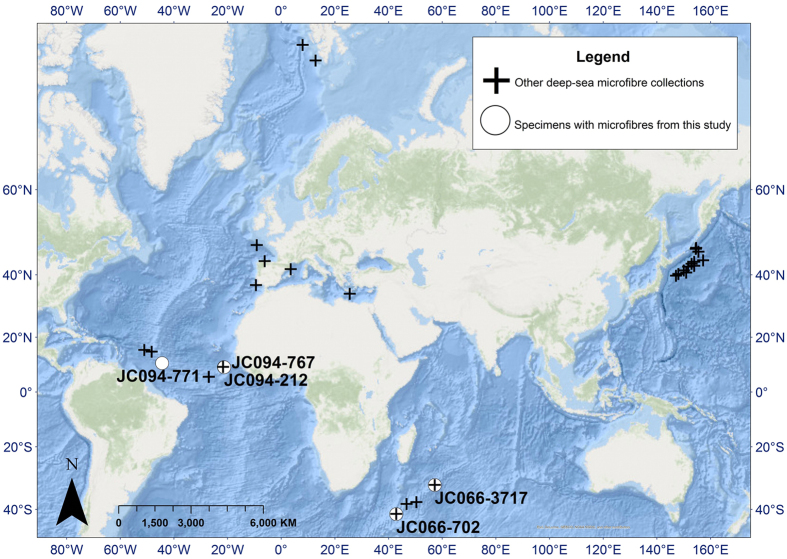
Map showing all known deep-sea microfibre collection locations (sediment cores from^25^, unpublished sediment core results from JC094, West Pacific microfibres from^35^, specimens – white circles - are from this study). Labels refer to specimen codes of organisms (see Table 1). Map made with ArcGIS v.10.3.1. http://desktop.arcgis.com/en/arcmap/.

The organisms were collected using the manipulator arm and suction hose of a remotely operated vehicle (ROV) on expeditions on the R.R.S. *James Cook* in the SW Indian Ocean (JC066 in 2011) and equatorial mid-Atlantic (JC094 in 2013) (Fig. 4). At the same time core-top sediment samples were collected and microplastics were also found in those sediments^25^.

Historically, collections of deep-sea organisms have been made using dredging/trawling equipment, so that the exact locations of sample collection were unknown. Dredge sampling also causes organisms to be in a highly disturbed condition on recovery at the sea surface and trawls are often made of plastic fibres precluding the study of plastic contamination. By using ROVs the exact location and habitat is known, as the collections are made using a suction hose or manipulator arm and deposited into sample containers (bioboxes – made of plastic but not of the type and colour found). These sampling methods limit the potential for contamination by surrounding sediments and reduce trauma, maintaining the structural integrity of organisms. There is potential for contamination when ascending to the surface in bioboxes as they have some seawater through flow. However this type of contamination (and feeding during ascent) is unlikely and should result in microfibres of similar compositions being found on the external surfaces of the organisms^37^, which was not observed. Preservation fluid (70–80% ethanol) was not filtered for microplastics however some organisms were dead (caused by the pressure and temperature change when moving from deep to shallow water) when preserved i.e. not feeding and no organisms were observed feeding once on ship.

Laboratory fibre contamination was minimised through a stringent set of protocols based upon known and accepted procedures used in forensic laboratories that examine fibres evidence^49^. All on-shore work was undertaken in a sealed room (with door covered by 100% cotton muslin cloth) where only natural fibre clothing and non-plastic equipment (metal and glass) were utilised; the room had been cleaned and was monitored for microplastics. Clean dampened filter paper was used to sample for any microfibres present in the room during specimen dissection (see Woodall, *et al*.^37^ for full laboratory procedures). No synthetic fibres were found on the filters in any part of the study.

Stomach, mouth, all internal cavities and breathing organs (gills and ventilation cavities) were dissected from nine deep-sea organisms and examined under a binocular microscope to identify whether or not they had ingested or internalised microplastics (Table 1). Material was placed into glass petri dishes that had been cleaned using 0.22 μm membrane filtered Millipore water (as was all equipment). Only the dish under the microscope was open to the air and nearby dampened filters were monitored post-dissection to check for contamination. All plastic fibres were picked up using a metal entomological pin, and placed into Millipore water contained in a small, clean, glass vial which was immediately sealed. These anti-contamination procedures have proven to effectively minimise fibre contamination and, although complete removal of fibres from an environment is not possible, the amount remaining is minimal, can be monitored, and is acceptable for the exacting standards of the criminal justice system^50^. Microfibres were classified using a Nikon polarised light microscope. This method is commonly used in forensic science and other polymer sciences and has proven benefits for the fast and effective identification of fibres. This method is described in Woodall *et al*.^37^.

## Additional Information

**How to cite this article**: Taylor, M. L. *et al*. Plastic microfibre ingestion by deep-sea organisms. *Sci. Rep*. **6**, 33997; doi: 10.1038/srep33997 (2016).

## Figures and Tables

**Table 1:** Organisms examined for microplastics.

Sample ID	Organism ID	Phylum	Organism Locality	Depth (m)	Fibre ID	Material	Class§
JC094-201	Anemone	Cnidaria	Equatorial mid-Atlantic	836	None	N/A	N/A
JC094-224	Armoured holothurian (sea cucumber)	Echinodermata	Equatorial mid-Atlantic	671	None	N/A	N/A
JC066-3155	Octocoral - *Anthomastus*	Cnidaria	SW Indian Ocean	562	None	N/A	N/A
JC094-212	Holothurian (sea cucumber)	Echinodermata	Equatorial mid-Atlantic	334	212-1	Natural	
					212-2	Modified Acrylic	1
					212-3	Natural	
					212-4	Cotton	
					212-5	Cotton	
					212-6	Polyprop¶	1
JC094-771	Squat Lobster	Arthropoda	Equatorial mid-Atlantic	611	771-1	Natural	
					771-2	Viscose	1
					771-3	Cotton	
					771-4	Polyester	4
					771-5	Viscose	2
					771-6	Natural	
*JC094-767	Zoanthid on bamboo coral	Cnidaria	Equatorial mid-Atlantic	1783	767-1	Viscose	4
					767-2	Natural	
					767-3	Natural	
JC066-3717	Sea pen (octocoral)	Cnidaria	SW Indian Ocean	954	3717-1	Viscose	3
					3717-2	Natural	
					3717-3	Natural	
					3717-4	Polyester	1
JC066-702	Hermit Crab	Arthropoda	SW Indian Ocean	1062	702-1	Acrylic	1
					702-2	Synthetic (nylon or polyethylene)	1
					702-3	Natural	
					702-4	Natural	
					702-5	Natural	
					702-6	Polyester	2
					702-7	Polyprop¶	2
					702-8	Acrylic	2
JC066-702	Zoanthid	Cnidaria	SW Indian Ocean	1062	702-9	Modified acrylic	2
					702-10	Polyester	3

^*^Fibres found on exterior of organism.

^§^ Fibre classes differentiated as in ref. 37 (Table S1).

^¶^polyprop is an abbreviation of polypropylene.
